# Hierarchical Carbon Network Composites Derived from ZIF-8 for High-Efficiency Microwave Absorption

**DOI:** 10.3390/ma16093380

**Published:** 2023-04-26

**Authors:** Zhongyi Luo, Zhaohao Wang, Jinshuai Liu, Huihui Jin, Chunhua Han, Xuanpeng Wang

**Affiliations:** 1 Department of Physical Science & Technology, School of Science, Wuhan University of Technology, Wuhan 430070, China; 2 School of Chemistry and Chemical Engineering, Hubei Polytechnic University, Huangshi 435003, China; 3 School of Materials Science and Engineering, Wuhan University of Technology, Wuhan 430070, Chinahch5927@whut.edu.cn (C.H.); 4 Hainan Institute, Wuhan University of Technology, Sanya 572000, China; 5 Hubei Longzhong Laboratory, Wuhan University of Technology (Xiangyang Demonstration Zone), Xiangyang 441000, China

**Keywords:** metal–organic framework, ZIF-8, carbon networks, microwave absorption performance

## Abstract

Metal–organic framework (MOF)-derived composites have gained wide attention due to their specific structures and enhanced performance. In this work, we prepared carbon nanotubes with Fe nanoparticles connected to two-dimensional (2D) hierarchical carbon network composites via a low-pressure gas–solid reaction strategy. Specifically, the three-dimensional (3D) networks derived from ZIF-8 exploited the carbon nanotubes with the function of charge modulation. Meanwhile, we utilized the interconnected 2D nanostructures to optimize impedance matching and facilitate multiple scattering, ultimately improving the overall microwave absorption performance. Furthermore, based on the well-designed structures, the composites prepared at 800 °C (Fe-N-C@CNTs-800) achieved the best reflection loss (RL) of −58.5 dB, thereby obtaining superior microwave absorption performance. Overall, this study provides a good groundwork for further investigation into the modification and dimension design of novel hierarchical microwave absorbers.

## 1. Introduction

Microwave absorption materials (MAMs) have become important to address the problems of electromagnetic pollution and transmission signal attenuation in practical applications due to the rapid adoption of communications systems [1,2,3,4]. To date, concerted efforts have been devoted to designing heterogeneous MAMs, such as carbon-based materials [5,6,7,8], magnetic metals [9,10], and conductive polymers [11]. However, the single attenuation mechanism and impedance mismatch of single-component absorbers have severely limited their practical application [12,13,14,15]. Hence, to overcome this limitation, the incorporation of ferromagnetic nanoparticles into carbon-based absorbers has exhibited great potential due to its synergetic mechanism [16,17].

Typically, metal–organic frameworks (MOFs) are potential precursors for ferromagnetic nanoparticles/porous carbon composites [18,19,20]. Recently, the development of MOF-derived MAMs has renewed research interest in microwave absorption mainly due to the tunable composition and diverse structures of the precursors [21,22,23]. Moreover, by selecting various organic ligands and metals, the final composition of MOF-derived absorbers may be adequately synthesized, thereby achieving excellent microwave absorption performance. For example, Cheng et al. [24] prepared hierarchical nano-spheres confined in carbon particles after pyrolyzing the bi-metal organic framework, which achieved a reflection loss of −32.43 dB at 9.19 GHz and an effective absorption bandwidth (EAB) of 4.2 GHz. However, although pyrolyzing MOFs is a convenient technique to achieve disparate ferromagnetic nanoparticles/porous carbon composited absorbers, the aggregation of nanoparticles from MOF precursors typically generates decreased specific surface areas [25]. Moreover, with the increase in reaction temperature, the collapse of carbon networks and pore structures often results in diminished microwave absorption capacity and extremely high fill loading [26,27,28].

Besides the selection of MOF precursors, rational dimension design is also essential in optimizing the electromagnetic wave (EMW) absorption performance of MOF-derived absorbers. As a rule, one-dimensional (1D) materials are suitable to construct efficient carrier transport paths in the microwave absorption field, while two-dimensional (2D) nanostructures play a crucial part in facilitating the multi-scattering of EMWs due to hierarchical structures [29]. Furthermore, the selection of the correct strategy in building the interconnected framework from different dimensional materials could be significant in promoting the microwave absorbing performance and effectively decreasing the filling ratio in the MAM field. Very recently, mix-dimensional MOF-derived structures, which combine the advantages of 1D and 2D materials, have drawn wide attention for use in microwave absorption [30]. For instance, Yang et al. utilized a carbonization method to prepare NiCo nanorod@CNT composites derived from MOF-74 under an Ar atmosphere [31]. Specifically, the outstanding minimum RL value of the composites was as high as −58.8 dB with an EAB of 6.5 GHz. Additionally, Huan et al. fabricated Co-NC@CF composites prepared using the ZIF@CF precursor to achieve efficient microwave absorption abilities through a well-designed process. After an autocatalytic pyrolysis process, the RL value of the composites could reach −50.1 dB and the corresponding EAB was 4.82 GHz [32]. Moreover, the mix-dimensional heterostructures may not have only cause multi-scattering inside the absorbers but also promoted impedance matching in the appropriate wave band [33]. However, to date, it is still challenging to design microwave absorbers with hierarchical structures using individual MOF precursors.

Therefore, in this work, we utilized a unique strategy to grow ZIF-8 coatings on Fe-doped ZnO surfaces using low-pressure vapor super-assembly [34]. After a series of controlled steps involving pyrolysis, template etching, and final annealing, the hierarchical Fe-N-C@CNTs composites with a remarkable microwave absorption ability were successfully prepared [19]. More specifically, the applicable Fe and N atoms were uniformly doped into 2D carbon layers, which led to various synergetic mechanisms. In addition, the elaborate template etching process vastly increased the porosity of the materials and the permittivity and impedance matching of the Fe-N-C@CNTs were regulated by in situ nanotubes. Overall, the 3D hierarchical Fe-N-C@CNTs composites exhibited enhanced absorption qualities (−58.5 dB) and a broad bandwidth (4.88 GHz). Therefore, this work provides a unique strategy for the design of the MOF-derived absorbers.

## 2. Materials and Methods

Ferric nitrate nonahydrate (Fe(NO_3_)_3_·9H_2_O, 99%), zinc acetate ((CH_3_COO)_2_Zn, 99%), citric acid monohydrate (99%), methanol (99%), and 2-methylimidazole (99%) were purchased from Sinopharm Chemical Reagent Co., Ltd. (Shanghai, China). All chemicals were analytical grade and were directly used as received.

**Figure 1:**
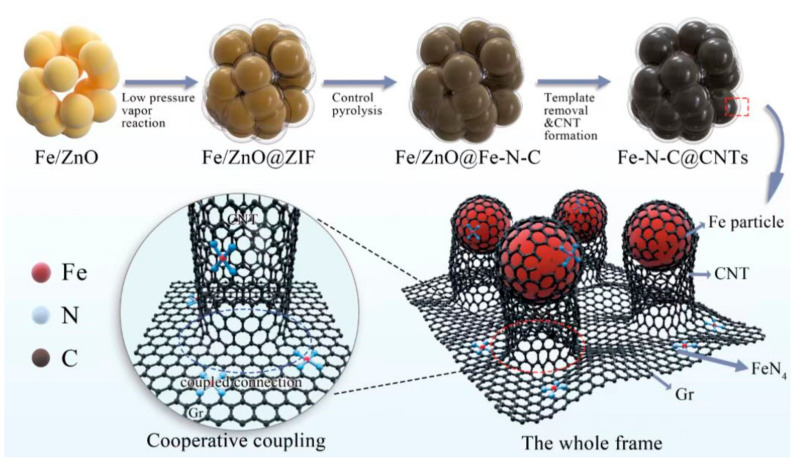
The description for synthetic process of Fe-N-C@CNTs composites.

The synthetic process of Fe-N-C@CNTs composites is shown in Figure 1. In this study, 18 mmol of zinc acetate and 2 mmol of ferric nitrate nonahydrate were dissolved in 170 mL of methanol at room temperature to obtain the mixed solution. Next, 40 mmol of citric acid monohydrate was dissolved in 50 mL of methanol and slowly added to the mixture. Specifically, a gel formed as the solution was dripped, and the gel was stirred for another 1 h to evenly disperse it. Following this, the beaker with the gel was inserted into an oven at 100 °C for 10 h. After this, the gel was heated to 600 °C at a rate of 5 °C/min in a tubular furnace for 6 h to obtain Fe-ZnO nanoparticles.

A porcelain boat loaded with 2 g of Fe-ZnO was transferred to a Petri dish filled with 2-methylimidazole. Next, the Petri dish with the sample was inserted into a vacuum drying oven at 140 °C and low pressure of about −100 Pa for 8 h to obtain the Fe-ZnO@ZIF composites. After this, the Fe-ZnO@ZIF composites were placed into a tubular furnace and heated to 600 °C at a rate of 5 °C/min in a nitrogen atmosphere for 2 h to realize the pyrolysis of ZIF on the surface and consequently, the Fe-ZnO@Fe-N-C with a core–shell structure was obtained. Further, the two-dimensional Fe-N-C structure was formed by etching the pyrolytic particles with dilute hydrochloric acid for 12 h to remove the Fe-ZnO core. Next, the excess heteroatoms were removed by centrifugation and washing three times. After this, the obtained Fe-N-C was heated to set temperature, in a mass fraction of 95% Ar and 5% H_2_ for 2 h. Finally, after the reaction was completed, the sample was retrieved to obtain the Fe-N-C@CNTs absorbers. The characterization of the materials is depicted in the Supplementary Materials.

The SEM images of the composites were measured using a scanning electron JSM-7100F microscope (JEOL, Tokyo, Japan) at an accelerating voltage of 25 kV. TEM images were collected using a JEM-2100F/Titan G2 60–300 microscope (JEOL, Japan). The XRD analysis of the crystal structure was performed using a smart lab diffractometer (Rigaku, Takatsuki, Japan) working at 30 kV and 10 mA with a Co Kα radiation source (λ = 1.79 Å). Raman spectroscopy and X-ray photoelectron spectroscopy were used to characterize the chemical states and composition of the samples. The electromagnetic parameters of standard coaxial ring samples mixed with paraffin (filler content was 20 wt.%) were recorded using a vector network analyzer (Agilent, N5244A, Keysight technologies, Santa Rosa, CA, USA).

## 3. Results

### 3.1. Morphologies and Microstructures

**Figure 2:**
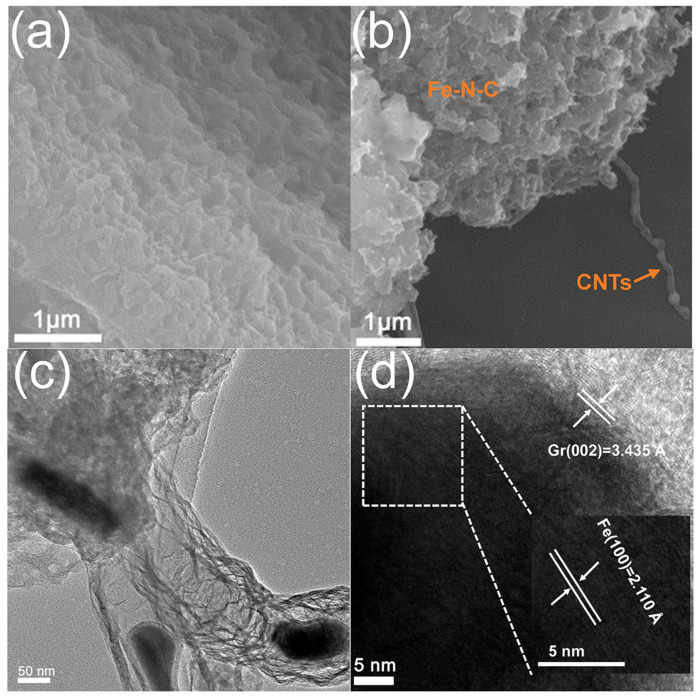
SEM image of (**a**) Fe-N-C, (**b**) Fe-N-C@CNTs-800. (**c**) TEM image, (**d**) HRTEM image of Fe-N-C@CNTs-800.

From the temperature adjustment of the gradient, it was found that the morphological structure at 800 °C was the best. Specifically, the scanning electron microscope (SEM) and transmission electron microscope (TEM) images revealed the microstructure and composition of the prepared material. As illustrated in Figure S1a, the TEM analysis of the Fe-ZnO@ZIF precursor revealed that the nanoparticles with sizes ranging from 200 to 500 nm retained their morphology and exhibited core–shell structures. Furthermore, the TEM images further confirmed the uniformity of the ZIF shell with a thickness of about 20 nm (Figure S1b,c). The corresponding energy dispersive spectrometry (EDS) elemental maps revealed the distribution of C, N, O, Fe, and Zn elements, which indicated the presence of these elements in the composite material (Figure S1d–f). Particularly, in Figure 2a, the Fe-N-C precursor is a typical 2D material and there were no carbon nanotubes. Meanwhile, the SEM images showed that Fe-N-C@CNTs-800 comprised carbon films stacked on top of each other, in which carbon nanotubes were grown. Additionally, the TEM images of Fe-N-C@CNTs-800 showed, with more clarity, that the synthesized wave-absorbing material was a hierarchical carbon film structure and the carbon film was connected with small carbon nanotubes, the top of which was coated with Fe nanoparticles (Figure 2b,c). Remarkably, almost all the iron nanoparticles were encapsulated in carbon nanotubes. More specifically, Figure 2d shows that the diameter of the Fe nanoparticles at the tip of the carbon tube was about 30 nm, which matches the diameter of the carbon tube, indicating that the mechanism of the Fe nanoparticles forming the carbon tube at high temperatures was the top growth mechanism. Moreover, the morphologies of Fe-N-C@CNTs-700 and Fe-N-C@CNTs-900 were also characterized (Figure S2). In particular, the corresponding images showed that the two samples had the structure of 1D carbon nanotubes coupled with 2D carbon films, and the Fe nanoparticles were at the top of the carbon nanotubes.

**Figure 3:**
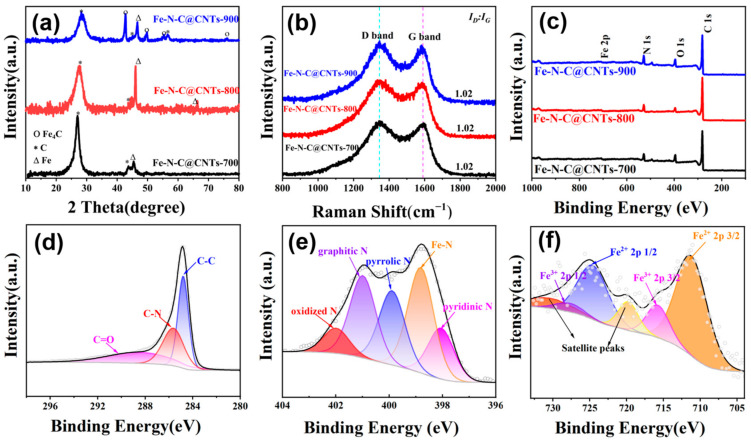
Elemental and phase composition analysis results: (**a**) XRD patterns, (**b**) Raman spectra, (**c**) XPS spectra of Fe-N-C@CNTs-700, 800, 900; high-resolution XPS spectra of (**d**) C 1s, (**e**) N 1s, (**f**) Fe 2p of Fe-N-C@CNTs-800 composites.

The powdered XRD of ZIF-8 and the comparison with Fe-ZnO@ZIF-8 is given in the Supplementary Materials (Figure S3). The X-ray diffraction (XRD) analysis spectra of these three materials (Figure 3a) showed that broad peaks were observed at 26.5° and 44.7°, which correspond to the (002) and (101) crystal planes of graphite carbon (No. 00-025-0284), respectively. In addition, the peak at 44.6° in the Fe-N-C@CNTs-700 spectrum corresponds to the γ (110) crystal plane of the phase Fe. In addition, from the Fe-N-C@CNTs-800 spectrum, the peak intensity was stronger and the two peaks at 44.6° and 65.0° were also attributed to the characteristic peaks of the phase Fe, which correspond to the (110) and (200) crystal planes, respectively (No. 03-065-4899). Meanwhile, the Fe-N-C@CNTs-900 showed sharper diffraction peaks near 40.2°, 46.8°, 52.7°, and 74.1°, matching the (111), (200), (210), and (300) crystal faces of Fe_4_C, respectively (No. 01-089-4053) except the characteristic peaks of the phase Fe and graphite carbon. Specifically, these characteristic peaks suggest that the type of the generated Fe particles also increased as the temperature rose [34]. Furthermore, the Raman spectra of all three samples showed the typical D peak band and G peak band of carbon materials (Figure 3b). Precisely, the ID/IG values of the three samples were approximate, indicating that the graphitization degree of the three samples was analogous [35]. Additionally, the XPS spectra were utilized to investigate and analyze the electronic structures of the elements in the three samples, and their spectra showed the characteristic peaks for Fe, N, and C (Figure 3c). Interestingly, it can be seen from Figure 3d that the C 1s spectrum of the composites was deconvoluted to three peaks—C-C, C-N, and C=O with a binding energy of 284.8, 285.7, and 289.1 eV, respectively [36]. Meanwhile, the N 1s spectrum could be deconvoluted into five peaks at 397.8, 398.6, 399.4, 400.8, and 402.9 eV, attributed to the pyridine-N, Fe-N, Pyrrole-N, graphite-N, and O-N, respectively (Figure 3e). Figure 3f displays the high-resolution Fe 2p spectrum, which indicates two Fe valence states [37]. Typically, the peaks were centered at 709.1 and 721.8 eV, which corresponds to Fe^2+^ 2p3/2 and Fe^2+^ 2p1/2, respectively, while the matching peaks of Fe^3+^ were attributed to 711.3 eV (2p3/2) and 724.6 eV (2p1/2), respectively. Moreover, it should be noted that the typical satellite peaks centered at 715.4 eV (2p3/2) and 729.2 eV (2p1/2) reflected the distinct spin-orbit couplets [38].

Furthermore, nitrogen adsorption–desorption isotherms were utilized to explore the specific surface area and pore structures. More precisely, as shown in Figure S4, the specific surface areas of Fe-N-C@CNTs synthesized at 700, 800, and 900 °C were 367.23, 283.93, and 222.15 m^2^ g^−1^, respectively. Specifically, the pore-size distribution diagram (inset of Figure S4) showed that there were more mesopores in these three samples, and with the increase in pyrolysis temperature, the pore size of the mesopores became smaller. This is presumably caused by the increasing amount of carbon nanotubes generated with increasing temperature [39]. The nitrogen adsorption–desorption isotherm curve of Fe-ZnO@ZIF-8 is shown in Figure S5. The BET surface area of Fe-ZnO@ZIF-8 was 304.22 m^2^/g, which was close to that of Fe-N-C@CNTs-700 (367.23 m^2^/g). In addition, the above-mentioned results revealed that with the increase in temperature, the pore size shrinks, but the degree of graphitization does not vary significantly.

### 3.2. Electromagnetic Parameters Analysis

**Figure 4:**
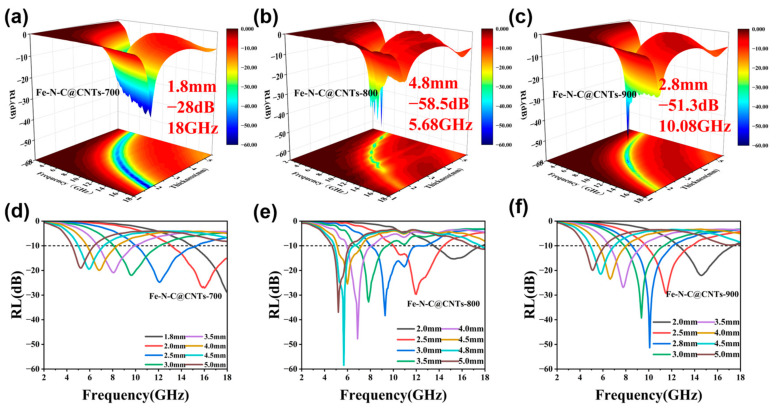
The reflection loss values and contour map with projection of (**a**,**d**) Fe-N-C@CNTs-700, (**b**,**e**) Fe-N-C@CNTs-800, (**c**,**f**) Fe-N-C@CNTs-900.

Consistent with the theory of transmission lines, the microwave absorption performance was evaluated via the values of the reflection loss (RL), which can be deduced by the formulas [40]:

(1) $$\begin{array}{l} RL=20\log_{10}\left|\frac{Z_{\mathrm{in}}-Z_{0}}{Z_{\mathrm{in}}+Z_{0}}\right| \end{array}$$

(2) $$Z_{\mathrm{in}}=Z_{0}{\left(\mu_{r}/\varepsilon_{r}\right)}^{1/2}\tanh\left[j\left(2\pi fd/c\right)\times {\left(\mu_{r}\cdot \varepsilon_{r}\right)}^{1/2}\right],$$

where *Z*_0_ is 377 Ω and *Z*_in_ is the input impedance. From 2.0 to 18.0 GHz, the RL plots of the as-prepared Fe-N-C@CNTs-700, 800, and 900 exhibited a frequency-dependent characteristic (Figure 4). Obviously, the three different absorbers had strong microwave absorbing capacities and excellent frequency responses. As functional MAMs, when the RL value is below −10 dB, it implies that more than 90% power of the EMW can be dissipated and the homologous frequency band is believed to be the effective absorber bandwidth (EAB). More precisely, the *RL_max_* value of Fe-N-C@CNTs-700 was −28 dB at 18 GHz with only 1.8 mm while the EAB was 6.24 GHz at 2.0 mm (Figure 4a,d). Meanwhile, the *RL_max_* value of Fe-N-C@CNTs-800 reached −58.5 dB when the thickness was 4.8 mm (Figure 4b). At a thickness of 3 mm, the EAB of Fe-N-C@CNTs-800 reached 4.88 GHz (Figure 4e). Specifically, the EAB of Fe-N-C@CNTs-800 could cover almost the whole X band, which is instructive for practical radar applications. Additionally, by controlling the final temperature, the *RL_max_* value of Fe-N-C@CNTs-900 reached −51.42 dB at 5.42 GHz and the EAB was 4.64 GHz at only a 2 mm thickness (Figure 4c,f). It is noteworthy that an unbefitting temperature may lead to the collapse of the carbon network, resulting in lower porosity and reduced microwave absorption performance.

**Figure 5:**
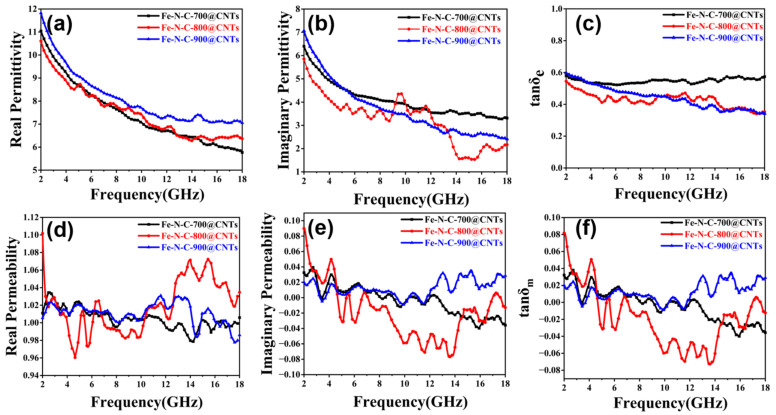
Electromagnetic parameters: (**a**) *ε*′, (**b**) *ε*″, and (**c**) tan*δ_ε_* of the as-prepared composites. (**d**) *μ*′, (**e**) *μ*″, and (**f**) tan*δ_μ_* of the as-prepared composites.

In order to verify the EMW absorption mechanisms of the composites with different annealing temperatures, the value of complex permittivity and permeability (*ε* = *ε*′ − *jε*″, *μ* = *μ*′ − *jμ*″) were measured using a network analyzer. It is well known that the EM parameters are crucial factors about microwave absorption performance. Generally, the real parts (*ε*′ and *μ*′) exhibit the capability of storing energy, meanwhile the imaginary parts (*ε*″ and *μ*″) represent the ability of dissipating microwave energy, respectively [41]. As shown in Figure 5, the *ε*′ values of specimens with a 20% mass ratio showed the tendency to decrease with increasing frequency in the range of 2–18 GHz, which exhibits a frequency-dependent characteristic. The values of *ε*′ decreased from 11.05 to 6.77 for Fe-N-C@CNTs-700, 10.61 to 6.37 for Fe-N-C@CNTs-800, and 11.82 to 7.05 for Fe-N-C@CNTs-900 (Figure 5a–c). Based on the charge transport theory, the value of *ε*′ and *ε*″ is higher and the conductivity of materials is higher. The *ε*″ values of Fe-N-C@CNTs-900 was always the highest among three samples, which reflects that the electromagnetic parameters could be significantly influenced by the pyrolysis temperature. As a whole, the imaginary permittivity also decreased in the range of 2–18 GHz. It is worth noting that the *ε*″ values of Fe-N-C@CNTs-800 presented multiple peaks in the frequency range of 4–18 GHz (Figure 5b). This could be the source of the electric polarization. The corresponding dielectric and magnetic loss ability of MAMs are usually measured using the dielectric and magnetic loss tangent (tan*δ_ε_* = *ε*″/*ε*′ and tan*δ_μ_* = *μ*″/*μ*′). The values of the dielectric loss tangent of Fe-N-C@CNTs-700 were stable in the range of 2–18 GHz. Meanwhile, the tan*δ_ε_* of Fe-N-C@CNTs-800 slightly fluctuated between 0.34 and 0.55 and the tan*δ_ε_* of Fe-N-C@CNTs-900 decreased from 0.60 to 0.34 with the frequency, which indicates satisfactory dielectric loss with electromagnetic waves among the three samples.

The *μ*′ of Fe-N-C@CNTs fabricated at 700 °C, 800 °C, and 900 °C fluctuated in the ranges of 0.98–1.03, 0.96–1.10, and 0.97–1.03, respectively. The carbon frameworks around the Fe particles might block the intergranular magnetic interaction [42]. In addition, the *μ*″ of Fe-N-C@CNTs-700, 800, and 900 also varied in the ranges of −0.04–0.04, −0.07–0.89, and −0.04–0.03, respectively (Figure 5d,e). Figure 5f shows the magnetic loss tangent of Fe-N-C@CNTs-700, Fe-N-C@CNTs-800, and Fe-N-C@CNTs-900. Overall, the three samples exhibited similar tendencies with their imaginary permeability in the range of −0.07–0.08. Specifically, it turns out that, for the attenuation of microwaves, the dielectric loss is critical.

### 3.3. Microwave Absorption Mechanism

**Figure 6:**
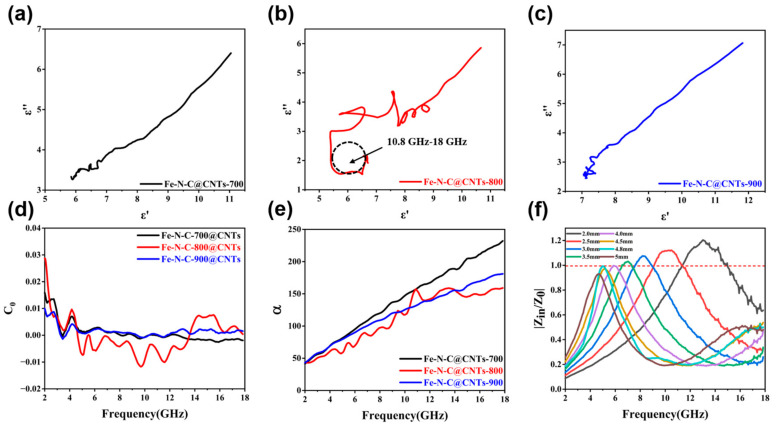
Cole–Cole curves: (**a**) Fe-N-C@CNTs-700, (**b**) Fe-N-C@CNTs-800, and (**c**) Fe-N-C@CNTs-900. (**d**) *C*_0_ and (**e**) *α* of as-prepared composites. (**f**) |*Z*_in_/*Z*_0_|of Fe-N-C@CNTs-800.

Besides the electrical loss, polarization loss is also significant for MAMs. Specifically, the Cole–Cole curves, which are derived from the Debye theory, describe the polarization relaxation from the following equation [43]:

(3) $${\left(\varepsilon^{\prime }-\frac{\varepsilon_{s}+\varepsilon_{\infty }}{2}\right)}^{2}+{\left(\varepsilon^{\prime \prime }\right)}^{2}={\left(\frac{\varepsilon_{s}-\varepsilon_{\infty }}{2}\right)}^{2},$$

where *ε_s_* and *ε*_∞_ represent the static dielectric constant and the dielectric constant at the frequency limit, respectively. Specifically, the polarization relaxation process is ensured if the curves of *ε*′–*ε*″ are semicircles. More precisely, the Cole–Cole curves of Fe-N-C@CNTs-700 and 900 were tiny and the Fe-N-C@CNTs-800 curves showed more than four obvious semicircles with the biggest semicircles ranging from 10.8 to 18 GHz, reflecting the existence of polarization relaxations (Figure 6a–c). In particular, the dipoles in the Fe-N-C@CNTs absorbers possessed extra polarization to promote the loss of microwave energy in the hierarchical carbon network. Meanwhile, the mechanism of specific magnetic loss was investigated from 2 to 18 GHz. Typically, the *C*_0_ value is usually utilized to justify the mechanism of eddy current loss, as calculated by the following formula [44]:

(4) $$C_{0}=\mu^{\prime \prime }{\left(\mu^{\prime }\right)}^{-2}f^{-1}$$

If *C*_0_ values are constant and independent from frequency, it reveals that the primary loss is from the eddy current. Conversely, natural resonance loss is the leading factor. From Figure 6d, all three samples exhibited similar fluctuations at 2.72 GHz and 4.16 GHz. Obviously, the *C*_0_ curve of Fe-N-C@CNTs-800 showed a more distinct peak in all the tested bands, which reflects a more natural resonance and exchange resonance. Furthermore, the attenuation constants (*α*) are essential to reveal the ability of the absorbers, which can be defined through the equation [45]:

(5) $$\alpha =\frac{\sqrt{2}\pi f}{c}\times \sqrt{(\mu^{\prime \prime }\varepsilon^{\prime \prime }-\mu^{\prime }\varepsilon^{\prime })+\sqrt{{\left(\mu^{\prime }\varepsilon^{\prime \prime }+\mu^{\prime \prime }\varepsilon^{\prime }\right)}^{2}+{\left(\mu^{\prime \prime }\varepsilon^{\prime \prime }-\mu^{\prime }\varepsilon^{\prime }\right)}^{2}}}$$

Figure 6e shows the calculated *α* values of the three samples that presented rising tendencies with increasing frequency. The Fe-N-C@CNTs-800 and Fe-N-C@CNTs-900 had moderate α values, while the Fe-N-C@CNTs-700 obtained a maximal attenuation constant. Subsequently, the normalized impedance *Z*(|*Z*_in_/*Z*_0_|) is also vital to assess the EMW absorption performance. According to Equation (2), if the values of |*Z*_in_/*Z*_0_| verge on 1, it indicates ideal impedance and better capacity to allow electromagnetic waves into absorbers [45]. In particular, the behavior of well-designed absorbers is due to the balance of high α value and good impedance match. The impedance matching of Fe-N-C@CNTs-800 with different thicknesses is shown in Figure 6f. More precisely, the red dashed curve marks |*Z*| = 1.0. It can be seen that the |*Z*| is close to 1 in the appropriate frequency.

Table S1 exhibits the microwave absorption performance of other recently reported absorbers. Obviously, compared to the related MOF-derived materials, Fe-N-C@CNTs absorbers show high-efficiency absorbing capabilities in effective bandwidths and maximum reflection loss.

## 4. Discussion

**Figure 7:**
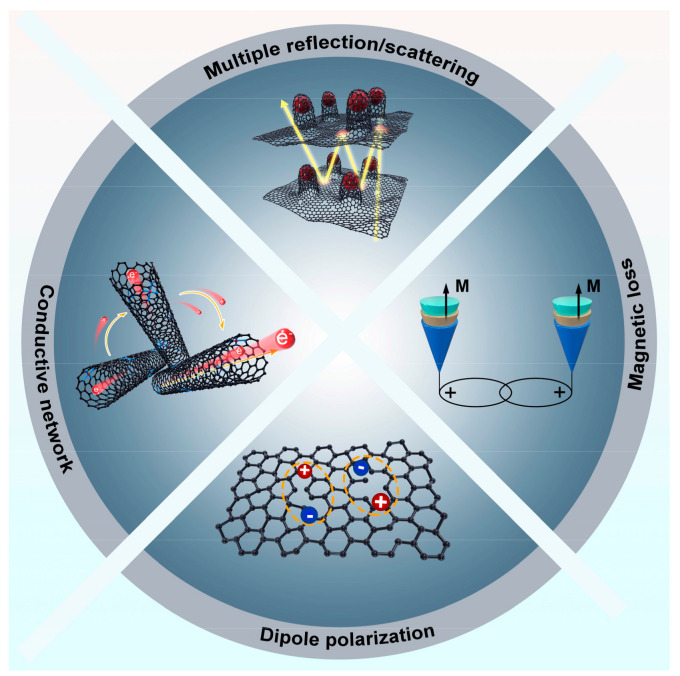
The potential absorbing mechanism of Fe-N-C@CNTs absorbers.

Figure 7 provides the potential attenuation mechanism of Fe-N-C@CNTs composites more intuitively. First, the outstanding performance is ascribed to the hierarchical structures derived by ZIF-8, which extends more transmission paths of incident EMWs between the different heterogeneous structures. Second, the conductive carbon network built by graphitized carbon and in situ-grown nanotubes could greatly optimize the conductivity of the absorbers and improve the transport of free electrons. Meanwhile, to increase the efficiency of converting the power of EMWs into thermal energy in the alternating EMW field, a microcurrent is generated by excited electrons among the interconnected carbon networks. Moreover, the addition of doped nitrogen atoms and defects caused by the template removal in the composites could be the source of polarization, which promotes microwave absorption performance. Finally, the distributed Fe nanoparticles between the CNTs and 2D carbon structures also significantly increase the magnetic loss in the composites. Therefore, the ZIF-8 derived hierarchical Fe-N-C@CNTs composites achieved enhanced microwave attenuation capacity owing to multiple synergetic mechanisms.

## 5. Conclusions

In this study, we utilized the low-pressure vapor assembly strategy and template method to fabricate Fe-N-C@CNTs composites. Specifically, the method is applicable in rational dimension design and opens new perspectives on microwave absorbers. By controlling the final pyrolysis temperature, in situ-grown CNTs provide more heterogeneous interfaces and optimize impedance matching. More precisely, we utilized a unique strategy to achieve the ZIF-8-derived absorbers, which exhibit a microwave absorption performance of −58.5 dB in the X band and the EAB reached 4.88 GHz at 3.0 mm. Overall, the study provides a unique strategy for the novel design of lightweight and efficient absorbers.

## Data Availability

Not applicable.

## Supplementary Materials

The following supporting information can be downloaded at: https://www.mdpi.com/article/10.3390/ma16093380/s1, Figure S1: (a,b) TEM images of Fe-ZnO@ZIF; (c–h) EDS mapping of Fe-ZnO@ZIF; Figure S2: TEM images of (a–c) Fe-N-C@CNTs-700; (d–f) Fe-N-C@CNTs-900; Figure S3: N_2_ adsorption–desorption isotherms of Fe-N-C@CNTs-700, 800, 900. Figure S4. N_2_ adsorption−desorption isotherms of Fe-N-C@CNTs-700, 800, 900. Figure S5. The Nitrogen adsorption-desorption isotherm curve of Fe-ZnO@ZIF-8. Table S1: Comparisons of microwave-absorption performance between our work and those from related works. References [46,47,48,49,50] are cited in the Supplementary Materials.
