# Supplementary material for: Hierarchical Carbon Network Composites Derived from ZIF-8 for High-Efficiency Microwave Absorption
Source: Materials (Basel). 2023 Apr 26;16(9):3380. doi: 10.3390/ma16093380 (PMC10180149; doi:10.3390/ma16093380)
Supplement: Supplementary file 1 [file materials-16-03380-s001.zip › materials-2310471-supplementary.pdf]

Supporting Information

Hierarchical Carbon Network Composites Derived from ZIF-8 for High-Efficiency Microwave Absorption

Zhongyi Luo [1], Zhaohao Wang [2], Jinshuai Liu [3,4], Huihui Jin [1,*], Chunhua Han [3,4,5] and Xuanpeng Wang [1,4,5,*]

[1] Department of Physical Science & Technology, School of Science, Wuhan University of Technology, Wuhan 430070, China
[2] School of Chemistry and Chemical Engineering, Hubei Polytechnic University, Huangshi 435003, China
[3] School of Materials Science and Engineering, Wuhan University of Technology, Wuhan 430070, China
[4] Hainan Institute, Wuhan University of Technology, Sanya 572000, China
[5] Hubei Longzhong Laboratory, Wuhan University of Technology (Xiangyang Demonstration Zone), Xiangyang 441000, China
* Correspondence: jinhuihui@whut.edu.cn (H.J.); wxp122525691@whut.edu.cn (X.W.); Tel.: +86-135-4528-4506 (X.W.)

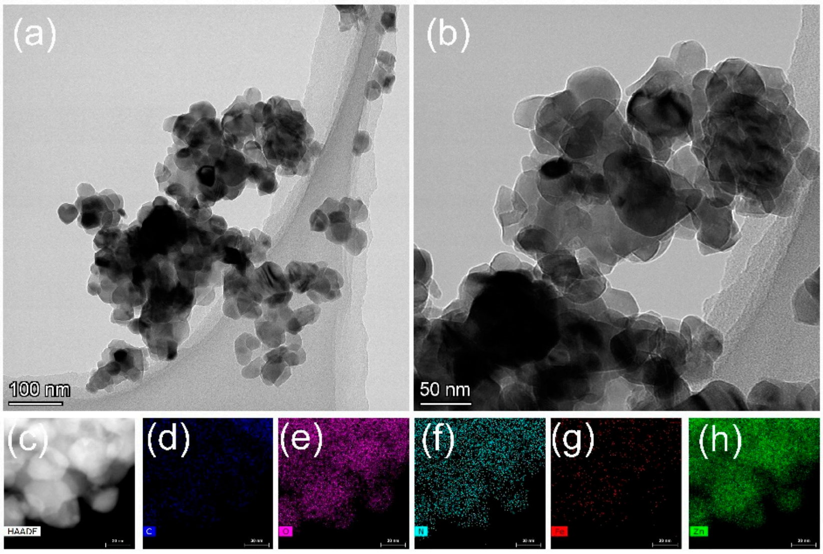

Figure S1. (a,b) TEM images of Fe-ZnO@ZIF; (c–h) EDS mapping of Fe-ZnO@ZIF.

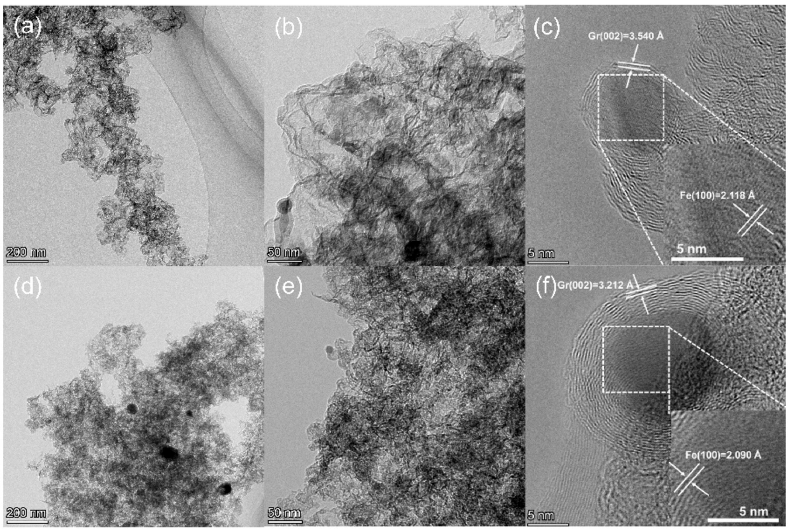

Figure S2. TEM images of (a–c) Fe-N-C@CNTs-700; (d–f) Fe-N-C@CNTs-900.

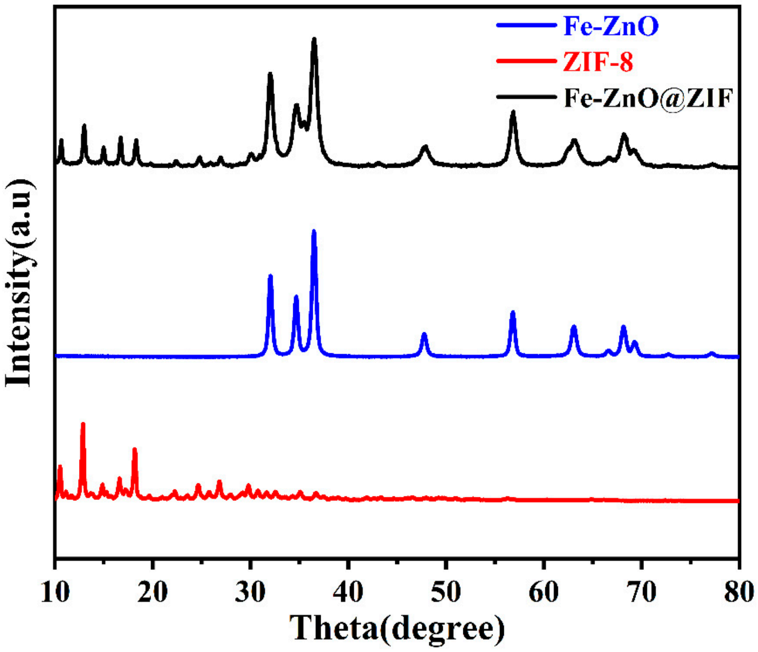

Figure S3. The powdered XRD of ZIF-8, Fe-ZnO@ZIF-8, and Fe-ZnO.

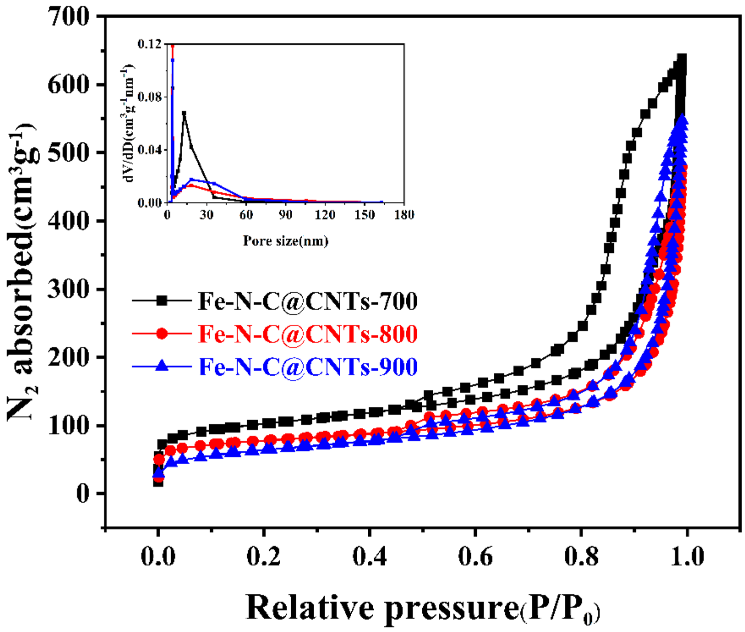

Figure S4. N_2 adsorption−desorption isotherms of Fe-N-C@CNTs-700, 800, 900.

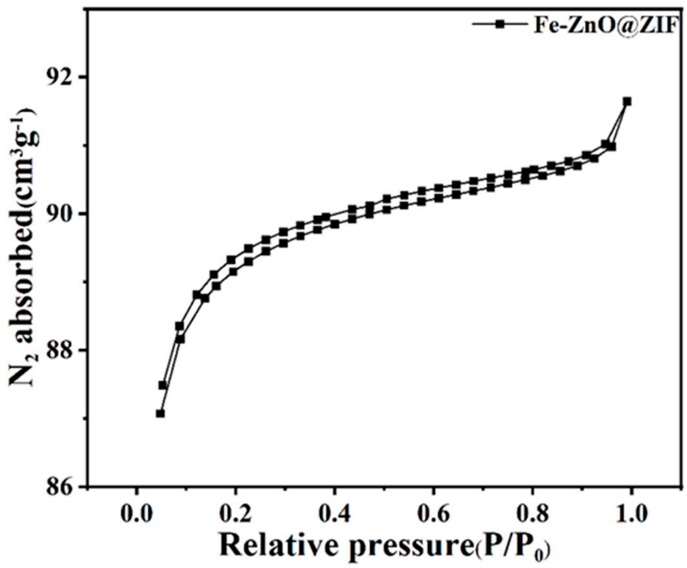

Figure S5. The Nitrogen adsorption-desorption isotherm curve of Fe-ZnO@ZIF-8.

Table S1. Comparisons of microwave-absorption performance between our work and related literatures.

Compositon	RL (dB)	EAB (GHz)	Thickness (mm)	Filling Ratio (wt.%)	Reference
Fe-N/C	-30.98	5.04	1.7	33.3	[46]
Co/N/C	-27.2	5.97	6.68	15	[47]
CoNC/CNTs	-44.6	1.7	4.7	15	[48]
Ni@C@ZnO	-55.8	4.1	2.5	25	[49]
CoFe@C	-44.1	5.2	5.8	40	[50]
Fe-N-C@CNTs	-58.5	5.68	4.8	20	This work
